# Adiponectin and Inflammatory Marker Levels in the Elderly Patients with Diabetes, Mild Cognitive Impairment and Depressive Symptoms

**DOI:** 10.3390/ijms251910804

**Published:** 2024-10-08

**Authors:** Malgorzata Gorska-Ciebiada, Maciej Ciebiada

**Affiliations:** 1 Department of Propaedeutics of Lifestyle Diseases, Medical University of Lodz, 90-251 Lodz, Poland; 2 Department of General and Oncological Pneumology, Medical University of Lodz, 90-549 Lodz, Poland; maciej.ciebiada@umed.lodz.pl

**Keywords:** adiponectin, hs-CRP, TNF-α, mild cognitive impairment, depressive symptoms, elderly, type 2 diabetes

## Abstract

Some studies suggest that low-grade inflammation and adipokines may be involved in mild cognitive impairment (MCI) and depression in subjects with type 2 diabetes; however, the available data concerning the elderly population are limited. Therefore, we conducted novel research to determine the serum adiponectin, hs-CRP and TNF-α levels in elderly diabetic patients with MCI and depressive symptoms and to identify the factors associated with MCI in this group. A total of 178 diabetic patients (mean age 84.4 ± 3.4 years) were screened for MCI and depressive symptoms. Various biochemical and biomarker data were collected. We found that patients with MCI and depressive symptoms demonstrated lower adiponectin levels and high hs-CRP and TNF-α. In this group, adiponectin concentration was negatively correlated with hs-CRP, TNF-α, HbA1c, and GDS-30 scores and positively correlated with MoCA scores. Multivariable analysis found the risk of MCI to be associated with higher TNF-α levels, fewer years of formal education, an increased number of comorbidities, and the presence of CVD. We concluded that low-grade inflammation and the presence of adipokines are associated with MCI and depressive symptoms in elderly diabetics. Further research should evaluate the suitability of Hs-CRP, TNF-α, and adiponectin as diagnostic markers for MCI and potential therapeutic targets.

## 1. Introduction

The global population is increasingly ageing, resulting in a higher prevalence of many chronic diseases, which presents a significant burden for public health systems. As life expectancy increases, so does the occurrence of dementia, diabetes, and depression, which affects the well-being of the elderly, their families, and society as a whole.

The International Diabetes Federation (IDF) predicts that the number of adults with diabetes worldwide will rise from 537 million in 2021 to 783 million by 2045 [1]. Diabetes is most prevalent in the senior population, reaching 29.2% among those aged 65 years and older (16.5 million seniors) in the United States [2]. Diabetes through chronic hyperglycemia, insulin resistance, and vascular damage is a well-known risk factor for cognitive decline and dementia in late life [3]. It is preceded by mild cognitive impairment (MCI), characterized by more severe cognitive changes than those associated with normal ageing but which do not interfere with daily life [4]. Long-term prospective epidemiological studies suggest that MCI progresses to dementia in 10–15% of cases annually [5]. Diabetes is also described as a risk factor for depression [6,7]. The risk of depression is also increased by diabetic complications, comorbidities, poorer physical condition, lower social and family support, economic problems, and, finally, old age with lower quality of life [8]. Taking into account the frequent co-occurrence of diabetes, depression, and dementia, it has been suggested that all three conditions may share a common pathophysiological background.

It has been proposed that these conditions could all be associated with microvascular dysfunctions in the diabetic brain; such problems are driven by hyperglycaemia, obesity, hypertension, and insulin resistance and lead to an increased risk of cognitive impairment, depression, and stroke [9]. The effect of cerebral microvascular dysfunction in diabetes is still under investigation. This pathology is related to increased oxidative stress, inflammatory responses, and disturbed blood–brain barrier permeability. This results in leakage of proteins and other mediators into the perivascular space. Microvascular dysfunction also leads to changes in perfusion, hypoxia, and increased angiogenesis [9]. Also, some inflammatory processes could support the development of cognitive dysfunction through the intensification of cerebral small vessel disease, atherosclerosis, and endothelial damage [10].

Low-grade chronic inflammation has been widely described in depression. It is characterized by elevated levels of cytokines such as tumour necrosis factor-α (TNF-α) and interleukin 6 (IL-6), as well as C-reactive protein (CRP) [11,12]. Persistent hyperglycemia in diabetes leads to increased production of advanced glycation end products (AGEs), which, through activation of their receptors, may stimulate the whole inflammatory cascade. The effects of an elevated release of cytokines and reactive oxygen species include activation of microglia in the brain and microvascular damage. The neuroinflammation present in diabetes may also disturb monoamine metabolism and hypothalamic–pituitary–adrenal axis function, which are crucial in the pathogenesis of depression [11,12]. Central or abdominal obesity, frequently associated with diabetes and depression, favours the secretion of pro-inflammatory cytokines through activated adipocytes and macrophages, thus leading to the development of insulin resistance [13].

Some of the neurological dysfunctions occurring in the brain and the subsequent development of dementia, reported in subjects with depression, could be explained by changes in the brain energy metabolism, excessive oxidative stress, and chronic inflammation [14]. In addition, adiponectin, a hormone derived from adipose tissue, is reported to increase insulin sensitivity and lower systemic inflammation [15]. After crossing the blood–brain barrier, adiponectin can act as a neuroprotective and antidepressant molecule, with beneficial effects on hippocampal neurogenesis and cognitive function [16,17]. It has anti-inflammatory properties, which are manifested through the suppression of TNF-α synthesis and inhibition of TNF-α-mediated inflammatory signalling from macrophages [18]. In vivo and in vitro studies indicate that adiponectin can elevate the expression of anti-inflammatory markers, such as interleukin-10, and decrease the expression of pro-inflammatory cytokines, such as IL-6, TNF-α, and CRP [19]. The beneficial effects of adiponectin on the central nervous system are mostly described based on data from experimental studies [19]. For example, adiponectin-deficient mice displayed increased susceptibility to depressive behaviours [19]. Another study showed that adiponectin incubation improved neuronal survival, reduced expression of reactive oxygen species, and reduced caspase-3 activity in hippocampal neurones driven from rats [19]. Although experimental studies indicate the potential beneficial role of adiponectin in the brain, data from clinical studies gave contradictory results [20]. Both cross-sectional and prospective studies showed conflicting results regarding the association between adiponectin levels and incidence of MCI or Alzheimer Disease [20].

Taken together, although some studies suggest that low-grade inflammation and adipokines may be involved in cognitive impairment and depression in subjects with T2DM, the available data concerning the elderly population are limited. In addition, there is growing evidence that vascular and lifestyle-related factors can vary considerably in older individuals compared to younger ones, and the predictive model of dementia is more complex in late age [21]. Therefore, there is a need to explore the pathophysiological background of cognitive decline in patients with diabetes, especially in those with comorbid depression, and determine its risk factors. Such studies focusing on elderly individuals and their specific age-related problems can support the development of more effective preventive strategies that can reverse, delay, or slow the onset of the disease.

Therefore, we conducted novel research to determine the levels of serum adiponectin, hs-CRP, and TNF-α in elderly patients with diabetes, MCI, and symptoms of depression. The aim of the study was also to identify the factors (vascular, inflammatory, and metabolic) associated with MCI in this group.

## 2. Results

### 2.1. Clinical Characteristics and Metabolic Parameters

The baseline characteristics of the four groups are compared in Table 1. The ANOVA/Kruskal–Wallis test indicated that age, education, sex, and partner status differed significantly among the four groups, as did various clinical parameters: smoking, physical activity, diabetes duration, macrovascular and microvascular complications, hyperlipidemia, comorbidities, and anti-diabetic treatment. The multiple comparisons test also identified significant differences between the four groups with regard to BMI, HbA1c, lipids, GDS-30 score, and MoCA score.

The post hoc test showed that subjects with MCI tended to have a lower education level, a longer duration of diabetes, a higher number of co-morbidities, a higher level of HbA1c and triglycerides and lower concentrations of HDL cholesterol and MoCA scores compared to controls. They were also significantly more likely to be diagnosed with CVD, hyperlipidemia, retinopathy, and nephropathy.

Furthermore, the multiple comparisons revealed that compared to controls, patients with depressive symptoms were more likely to be female, smoke, and have less physical activity. They were also characterized by a longer duration of diabetes, higher BMI, higher number of co-morbidities, higher level of total and LDL cholesterol, and a higher GDS-30 score. They were also more likely to be diagnosed with hyperlipidemia or polyneuropathy and were more commonly treated with insulin and less with oral anti-diabetic drugs.

Lastly, compared to controls, individuals with MCI and depressive symptoms tended to be older, less educated, female, and single. They were also more likely to report a longer duration of diabetes, a higher number of co-morbidities, higher BMI and GDS-30 scores, and higher levels of HbA1c and triglycerides. They were often characterized by lower HDL cholesterol concentrations and MoCA scores and were more likely to be diagnosed with CVD, stroke/TIA, and hyperlipidemia. They were more frequently treated with insulin and less with oral anti-diabetic drugs.

Interestingly, the patients with both MCI and depressive symptoms were significantly older than the other groups, had a higher number of co-morbidities, and were more likely to be diagnosed with stroke/TIA.

Lastly, we didn’t notice any differences between the studied groups in parameters such as systolic or diastolic blood pressure and fasting glucose (MCI—136.4 ± 16.4 mm Hg, 74.6 ± 7.8 mm Hg, and 127.6 ± 24.4 mg/dL, respectively); MCI with depressive symptoms—134.6 ± 18.8 mm Hg, 75.7 ± 7.7 mm Hg, and 131.9 ± 31.1 mg/dL, respectively; depressive symptoms without MCI—137.5 ± 17.1 mm Hg, 74.7 ± 6.9 mm Hg, and 130.9 ± 28.2 mg/dL, respectively; and controls—137.4 ± 15.0 mm Hg, 75.8 ± 7.9 mm Hg, and 131.1 ± 23.3 mg/dL, respectively. The frequency of parameters such as hypertension, family history of diabetes, anti-hypertensive therapy, and lipid-lowering therapy was similar in each studied group: MCI—21 (43.8%), 36 (75%), and 38 (79.2%), respectively; MCI with depressive symptoms—9 (42.8%), 17 (80.9%), and 18 (85.7%), respectively; depressive symptoms without MCI—19 (54.3%), 25 (71.4%), and 26 (74.3%), respectively; and controls—45 (60.8%), 53 (71.6%), and 49 (66.2%), respectively.

#### Adiponectin, hs-CRP and TNF-α Levels

**Figure 1:**
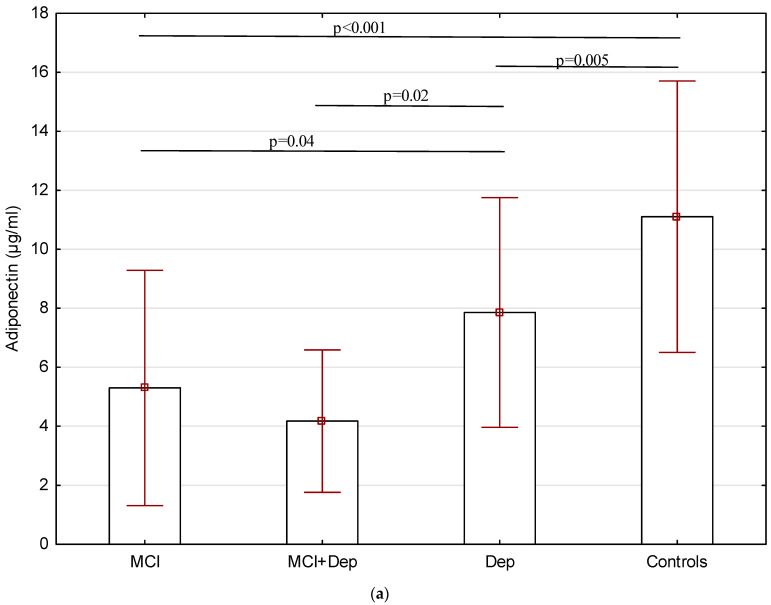

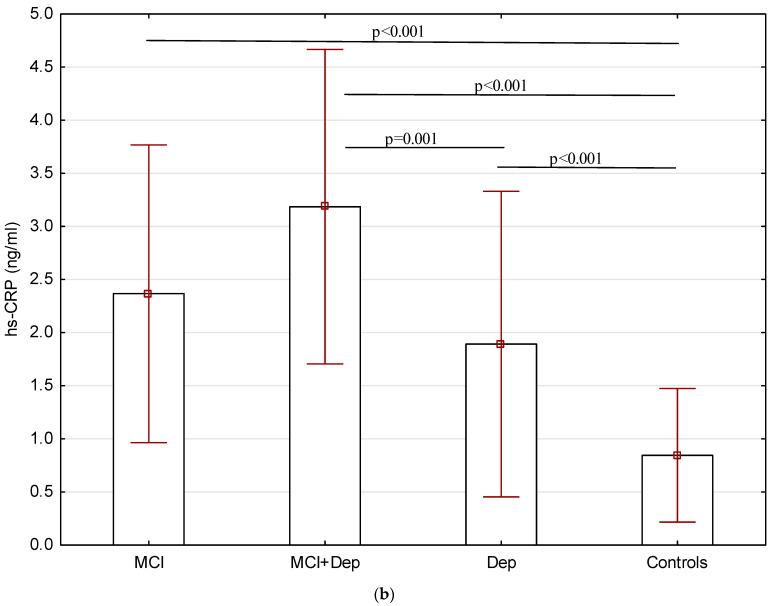

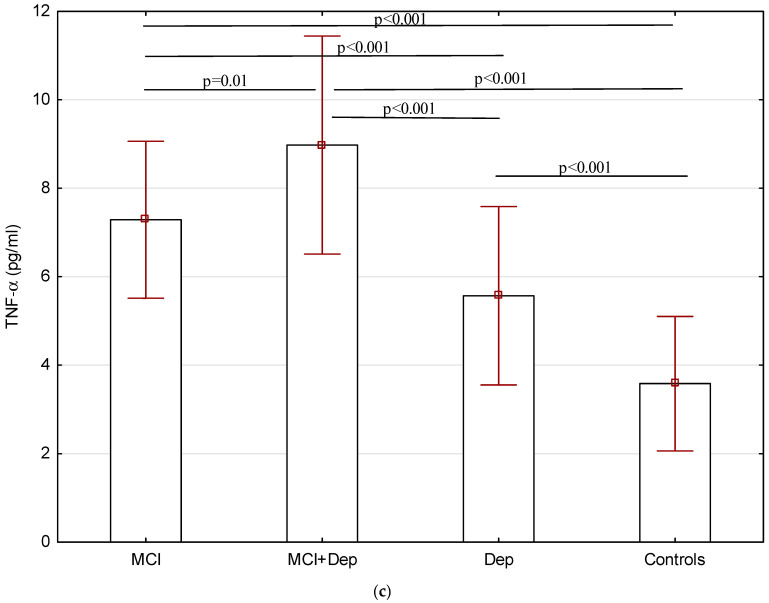
Serum levels of adiponectin (µg/mL) (**a**), hs-CRP (ng/mL) (**b**), and TNF-α (pg/mL) (**c**) in elderly patients with T2DM (MCI—mild cognitive impairment, Dep—depressive symptoms).

The analysis of variance (ANOVA) revealed that serum adiponectin, hs-CRP, and TNF-α levels differed significantly among the four groups (Figure 1a–c). The subjects with both MCI and depressive symptoms demonstrated the lowest levels of adiponectin (4.17 ± 2.4 µg/mL) and the highest concentrations of hs-CRP (3.18 ± 1.48 ng/mL) and TNF-α (8.97 ± 2.46 pg/mL). In contrast, the controls demonstrated the highest levels of adiponectin (11.1 ± 4.6 µg/mL) and the lowest concentrations of hs-CRP (0.84 ± 0.62 ng/mL) and TNF-α (3.58 ± 1.51 pg/mL). Compared to patients with depressive symptoms, the patients with MCI exhibited lower serum adiponectin levels (5.29 ± 3.9 µg/mL vs. 7.85 ± 3.8 µg/mL) and higher TNF-α (7.28 ± 1.7 pg/mL vs. 5.56 ± 2.0 pg/mL); they also had higher concentrations of hs-CRP, but this relationship was not significant (2.36 ± 1.4 ng/mL vs. 1.89 ± 1.4 ng/mL).

The correlation analyses are presented in Table 2. A significant positive correlation was found between hs-CRP and TNF-α, and both parameters were negatively correlated with adiponectin in all study groups. In subjects with MCI and depressive symptoms, strong inverse correlations (r > 0.7) were found between adiponectin concentration and hs-CRP, TNF-α, HbA1c, and GDS-30 scores, and a positive correlation between adiponectin levels and MoCA scores. Adiponectin concentrations were negatively correlated with BMI among the patients with MCI, those with depression, and in controls. The MCI group and the MCI with depressive symptoms group both demonstrated a positive correlation between hs-CRP and TNF-α concentrations, between hs-CRP and Hba1c levels, and between TNF-α and Hba1c levels. In the MCI patients, higher hs-CRP and TNF-α levels were associated with lower MoCA scores. Also, hs-CRP was correlated with MoCA scores in MCI subjects with comorbid depressive symptoms.

### 2.2. Risk Factor for MCI

The risk factors for MCI were identified by constructing univariate logistic regression models and then multivariable regression models (Table 3). The univariate logistic regression models found that a higher likelihood of diagnosis with MCI was associated with older age, fewer years of formal education, longer duration of diabetes, higher HbA1c, hs-CRP, TNF-α, and triglyceride levels and lower concentrations of adiponectin and HDL, as well as with the presence of CVD, hyperlipidemia, retinopathy, and nephropathy and a greater number of co-morbidities. Multiple logistic regression analysis also found that a greater chance of MCI in the studied patients was associated with a higher level of TNF-α, fewer years of formal education, increased number of co-morbidities, and the presence of CVD.

## 3. Discussion

The present study is the first to identify the factors associated with MCI in elderly diabetic subjects in Poland. It was found that the prevalence of MCI was 26.9%, the prevalence of depressive symptoms was 19.7%, and the prevalence of both MCI and comorbid depressive symptoms was 11.8%. A large meta-analysis comprising 17 studies containing the records of 4380 elderly subjects with diabetes found cognitive impairment to be present in 48% of participants and that it was more common in female patients who were older; it was also associated with a lower education level, the lack of a spouse, living alone, and a lower monthly income [22].

Many hypotheses have been proposed to explain the potential mechanisms underlying the interaction between depression, impaired cognition, and diabetes [23]. Firstly, late-life depression is connected with significant hippocampal volume loss and, thus, a greater risk of cognitive decline [24]. Secondly, depression symptomatology is often characterized by white matter hyperintensities and minor ischemic lesions, and these also contribute to multiple cognitive domain impairments, such as executive function, memory, global cognition, and processing speed [25]. Thirdly, depression may share some common mechanisms with dementia, such as altered amyloid-beta levels and metabolism, followed by early neurodegeneration [25].

The present study examined the associations between type 2 diabetes, depressive symptoms, and cognitive impairment in elderly patients. The relationships between these conditions appear to be complex and multifactorial; they may include shared risk factors and common mechanisms, such as low-grade inflammation. Our findings indicate that hs-CRP and TNF-α were highest in subjects with both MCI and depressive symptoms and lowest in controls. Also, TNF-α level and hs-CRP concentration were higher in patients with MCI compared to those with depressive symptoms, although not significantly. In addition, MCI patients and those with MCI and depressive symptoms demonstrated a positive correlation between hs-CRP and TNF-α levels, between hs-CRP and Hba1c levels, and between TNF-α and Hba1c levels. In MCI patients, higher hs-CRP and TNF-α levels were associated with lower MoCA scores. We observed this correlation considering hs-CRP and MoCA scores in MCI subjects with comorbid depressive symptoms. Finally, in the MCI patients, univariate analysis found hs-CRP and TNF-α to be significantly higher, while multivariate logistic regression found TNF-α concentration to be higher; these could be used as diagnostic indicators.

Our results are consistent with other observational studies performed on younger populations [26]. A recently published systemic review and meta-analysis encompassing 7483 patients with T2DM from 32 studies found MoCA scores to be significantly moderately negatively correlated with IL-6, TNF- α, and CRP levels [26]. The authors also found a positive correlation between CRP, TNF-α levels, and HbA1c, between TNF-α levels and fasting blood glucose (FBG), and between CRP and total cholesterol concentrations [26]. They concluded that those inflammatory markers are related to cognitive impairment in subjects with diabetes.

Chronic low-grade inflammation is involved in the development of T2DM, cognitive impairment and depression. Some mechanisms were proposed to evaluate these connections [9]. Hyperglycemia, obesity, insulin resistance, hypertension, and arterial stiffening could contribute to cerebral microvascular dysfunction [9]. High glucose concentration followed by dysregulation of glucose transport may induce dysfunction of endothelial cells, pericytes, and astrocytes in the brain. This is manifested through increased permeability of endothelium and procoagulant activity or higher leucocyte adhesion. Chronic hyperglycemia through increased production of AGEs induces an inflammatory response [9]. Insulin resistance in the brain enhances oxidative stress and mitochondrial dysfunction and impairs neuronal function [9].

Our findings indicate that higher levels of hs-CRP and TNF-α were associated with the presence of depressive symptoms in the tested diabetic subjects. Similar to our results, the authors report that patients with T2DM and comorbid depression had elevated levels of CRP and IL-6 compared to those diabetic subjects without depression [27].

The mechanism underlying depression in patients with diabetes is still under investigation; however, it may partly be explained by the development of insulin resistance in the brain, caused by a failure of neurons to respond normally to insulin [28]. This could be caused by increased activity of the hypothalamic–pituitary–adrenal (HPA) axis, reduced volume of the anterior cingulate cortex or hippocampal grey matter, or alterations in the reward system of the brain [28].

In our study, we found that compared to controls, individuals with MCI and depressive symptoms were more likely to be diagnosed with CVD and stroke/TIA; they were also significantly older, with a higher number of co-morbidities. This is consistent with previous observations [29] that at an advanced age, vascular damage is aggravated by the coexistence of diabetes and may favour the development of cognitive impairment. The presence of depression, caused by higher levels of pro-inflammatory factors and dysregulation of the HPA axis, can worsen endothelial dysfunction and increase the risk of vascular dementia and Alzheimer’s disease. In addition, depression appears to be commonly associated with living alone, unhealthy life habits, poorer control of diabetes, and increased risk of microvascular and macrovascular complications; as such, it may further aggravate cognitive decline. The links between diabetes, cognitive impairment, and depression may include also include shared risk factors such as social status, physical activity, lifestyle and nutrition, and chronic stress. Data from intervention studies showed that a healthy lifestyle (weight loss and regular exercise) may improve white matter hyperintensities and ventricle volume, with beneficial effects on depression [30]. On the other hand, physical exercise or antidepressant treatment could improve hippocampal neurogenesis, which is affected by many factors, such as ageing and stress [31].

In the present study, individuals with MCI and depressive symptoms were older and had lower education levels than controls; they were also more likely to be female. Women are more sensitive and are prone to depression and anxiety. A lower education level can lower socioeconomic status and cause feelings of worthless and despair. Comorbidities may create additional burdens on patients and reduce their quality of their life.

Our data indicate that adiponectin levels were lowest in subjects with MCI and depressive symptoms and highest in controls. Serum adiponectin level was also lower in patients with MCI compared to those with depressive symptoms. In subjects with MCI and depressive symptoms, adiponectin concentration demonstrated strong inverse correlations with hs-CRP, TNF-α, HbA1c, and GDS-30 scores and a positive correlation with MoCA score. Adiponectin concentrations were negatively correlated with BMI in the MCI group, depressive subjects, and controls. Our results are consistent with other studies which reported that adiponectin may be associated with depression [32,33,34]. Most studies indicate that patients with depression present lower levels of adiponectin, regardless of the type of depression [32,33,34]. A systematic review and meta-analysis revealed that older subjects with BMI ≥ 25 and diagnosed with major depressive disorder presented lower adiponectin concentrations compared to those without major depressive disorders [34]. In contrast, one longitudinal study with elderly subjects showed that increased adiponectin levels and lower BMI were both associated with worsening depressive symptoms [35]. These discrepancies between our study and other data may result from the influence of various factors such as age, BMI, comorbidity, treatment, other population composition, and some methodological differences.

In one study that analyzed T2DM and T1DM separately, the authors showed that lower adiponectin levels and increased hs-CRP, IL-18, and IL-1RA concentrations were associated with higher depressive symptoms [36]. These associations were independent of multiple confounders. These are in line with our present findings: adiponectin concentration demonstrated a strong inverse correlation with hs-CRP and TNF-α in subjects with MCI and depressive symptoms. Thus, our findings support the hypothesis that the presence of depressive symptoms and MCI in older patients with diabetes may be exacerbated by the presence of more pronounced low-grade inflammation and adipokine dysregulation. Indeed, adiponectin is widely described as having anti-inflammatory effects [19]. In depression, adiponectin may reduce activation of the HPA axis by suppressing TNF-α production [19].

However, the data regarding the possible association between adiponectin levels in MCI subjects and cognitive decline and progress to dementia are conflicting. Similar to our results, Liu et al. found lower adiponectin levels in elderly MCI patients with T2DM compared to non-MCI diabetic and healthy controls [37]. The authors propose that age, lower education level, disease course of hypertension, disease course of diabetes, and a low adiponectin concentration may be risk factors for cognitive impairment. Another study also revealed lower levels of adiponectin and elevated CRP concentrations in adults with diabetes and cognitive impairment [38].

Little data exists about the role of adiponectin in the pathogenesis of dementia in diabetes, and even less regarding the role in MCI or dementia subjects. Some authors propose that serum adiponectin levels are lower in MCI and dementia patients [39]; however, other data indicate the presence of elevated concentrations in MCI [40]. Furthermore, many authors suggest that adiponectin may have beneficial effects on cognition by reducing the inflammation cascade, reducing amyloid beta production, and improving insulin sensitivity in the brain [18,19]. In addition, adiponectin demonstrates neuroprotective properties and could influence hippocampal neurogenesis and synaptic plasticity [19].

Our data are the first to indicate that inflammatory mediators and adiponectin levels may be associated with cognitive impairment and depressive symptoms in very old patients with diabetes. However, they have some limitations. Firstly, they are based on a relatively small sample with a certain selection bias. As such, further multi-center studies are needed with larger sample sizes to verify the results. In addition to the small sample, due to the specificity of the population (Polish diabetic patients over 80 years old, overweight women), the results cannot necessarily be generalized to other demographic groups or regions. Secondly, the age group used in the study was selected as ages 80 years or older, as indicated by the American Geriatric Society and the World Health Organization. It is possible that with the increase in life expectancy in developed countries, studies perhaps should consider including participants over 90 years of age. Even so, in such cases, the data should be interpreted with caution due to the presence of many confounders; for example, such older patients may have developed resilience to some of the risk factors for MCI or depression, which increases the risk in younger subjects. Lastly, as the study has a cross-sectional design, the results indicate only the correlations between some markers and the coexistence of cognitive impairment and depression in elderly patients with diabetes, so they are not suitable for proving cause-and-effect relationships. Further longitudinal studies with long-term follow-up and observation are needed to determine how low-grade inflammation and adipokine levels contribute to mood disorders and cognitive decline in diabetics in this elderly age group.

## 4. Materials and Methods

### 4.1. Study Population

The study was cross-sectional in design. A total of 178 T2DM patients, mean age 84.4 ± 3.4 years, (75 men, mean age 84.4 ± 3.5 years and 103 women, mean age 84.4 ± 3.4 years,), were recruited from an outpatient diabetes clinic affiliated with University Hospital no 1 in Lodz, Poland. Enrollment was consecutive with the following inclusion criteria: diagnosis with type 2 diabetes at least one year before inclusion in the study, age 80 and above, and full ability to understand and cooperate with study procedures. The subject was excluded if any of the following criteria were fulfilled: diagnosed with major depression or dementia, psychotic disorder, current use of antidepressant or antipsychotic medication, brain injuries (traumatic), substance use disorders (ethanol consumption, drug abuse), severe somatic illness (cancer), active infectious disease (COVID, influenza), or severe visual or hearing loss.

Each participant was assigned an identifying number to ensure anonymity.

Patients were classified into subjects with MCI (n-48), those with both MCI and depressive symptoms (n-21), or those with depressive symptoms (n-35).

Seventy-four diabetic subjects without MCI or depressive symptoms were recruited as controls.

### 4.2. Procedure

Prior to enrollment, the purpose, nature, and potential risks of the experiments were fully explained to the subjects, and each participant provided written consent to participate. The study protocol was approved by the local ethics committee and was performed in accordance with the Declaration of Helsinki.

After an initial screening assessment, all patients underwent a detailed interview that included a complete medical history, clinical diagnosis, and the applied treatment. All subjects then underwent a full physical examination, including measurement of body mass index (BMI) and blood pressure. Blood samples were obtained the next morning between 7 a.m. and 9 a.m. after an overnight fast. Following the samples, the participants were provided snacks to prevent hypoglycemia at the time of psychiatric assessment.

### 4.3. Assessment of MCI or Depressive Symptoms

Finally, all patients underwent neuropsychological examinations in a calm, quiet room to establish or rule out a diagnosis of MCI or depressive symptoms.

Various cognitive domains were assessed using the Montreal Cognitive Assessment (MoCA): visuospatial/executive function, naming, attention, abstraction, language, delayed memory, and orientation [41]. The total score ranges from 0 to 30, with lower scores indicating worse cognitive function. A MoCA score < 26 was assumed to indicate MCI. Although the level of education is a known factor that can influence the screening diagnostic test, the MoCA result is correlated with years of formal education. Therefore, subjects with less than 12 years of education level are awarded one extra point. The MoCA test is considered to have a higher sensitivity of 80.48% (compared to MMSE—66.4% sensitivity) and a better specificity of 81.19% (compared to MMSE—72.94% specificity) to detect MCI, and it is regarded as the best tool for screening cognitive impairment in the elderly population with diabetes [42,43].

All MCI patients were diagnosed based on the criteria proposed by the MCI Working Group of the European Consortium on Alzheimer’s Disease in 2006. These criteria comprise the following: cognitive complaints from the patient or family of the patient, a reported decline in cognitive function relative to that in the past year by the patient or guardian of the patient, cognitive disorders as evidenced by a clinical evaluation (impairment in memory or other cognitive domains), absence of major repercussions in activities of daily living, and absence of dementia (based on the Diagnostic and Statistical Manual of Mental Disorders-IV criteria) [44].

Depressive symptoms were assessed using the long version of the Geriatric Depression Scale (GDS-30) [45]. This validated questionnaire for the elderly population contains 30 items. Scores ranging from 0 to 9 are considered normal, and 10 to 19 are indicative of symptoms of depression. A score of 20 or above is accepted as an indicator of the presence of depression; any patients in this category were excluded from the study as demonstrating severe depressive symptomatology and referred to a psychiatrist for further diagnosis.

### 4.4. Definitions of Clinical Parameters

A set of demographic data was obtained from individuals at admission: age, education, sex, marital status, and clinical parameters, including smoking, physical activity, diabetes duration, family history of diabetes, and comorbidities. The body mass index (BMI) was calculated as weight (kilograms) divided by height squared (square meters). Blood pressure was measured with a standard mercury sphygmomanometer using the right arm in a seated position; two measurements were taken after a 10 min rest and averaged. Any history of previous cardiovascular disease or stroke/TIA, microvascular complications, e.g., presence of retinopathy, nephropathy, or polyneuropathy, was taken from medical records as well as the treatment. Two blood pressure measurements ≥ 140/90 mmHg or the use of any anti-hypertensive agents were considered as hypertension. Hyperlipidemia was defined as an untreated triglyceride level of 1.7 mmol/L or/and serum LDL cholesterol level of 2.6 mmol/L or receiving lipid-lowering treatment.

### 4.5. Serum Sample Collection and Laboratory Analysis

Blood samples were obtained for further analyses. Glycosylated haemoglobin (HbA1c), total cholesterol, triglyceride, low-density lipoprotein cholesterol (LDL), and high-density lipoprotein cholesterol (HDL) levels were measured in a central laboratory using standard methods.

### 4.6. Enzyme-Linked Immunosorbent Assay (ELISA)

In addition to routine laboratory testing, the serum concentrations of adiponectin, hs-CRP, and TNF-α were analyzed. A partial serum blood sample for each subject was obtained after centrifugation and then stored at −80 °C. The measurements were performed after all patients had been included in the study. The serum adiponectin (Human total Adiponectin/Acrp30, DRP300, R&D Systems (Bio-techne, Minneapolis, MN, USA), serum TNF-α levels (Human TNF-α, DTA00C, R&D Systems (Bio-techne, Minneapolis, MN, USA), and serum hs-CRP (hs-CRP ELISA kit, E0821h, EIAab, Wuhan, China) were measured using a commercially available Quantikine Human Immunoassay (ELISA) kit according to the manufacturer’s instructions. The detection ranges of the enzyme-linked immunosorbent assays (ELISA) were 0.079–0.891 ng/mL for adiponectin, 0.5–5.5 pg/mL for TNF-α, and 0.156–10 ng/mL for hs-CRP. All serum samples were assayed in duplicate, and samples for adiponectin were diluted 100 times. The intra-assay coefficient of variation for adiponectin was 4.7%, and the inter-assay coefficient of variation was 6.9%. For TNF-α, the intra-assay coefficient of variation was 4.6%, and the inter-assay coefficient of variation was 5.4%. For hs-CRP, the intra-assay coefficient of variation was 4.3%, and the inter-assay coefficient of variation was 6.4%. The minimum detectable concentrations were 0.246 ng/mL for adiponectin, 1.6 pg/mL for TNF-α, and 0.078 ng/mL for hs-CRP.

### 4.7. Statistical Analysis

Quantitative variables are described as means and standard deviation, and qualitative variables are described as counts and percentages. The Shapiro–Wilk test was used to confirm that the data had a normal distribution. Continuous variables were compared between the four groups using analysis of variance (ANOVA) followed by Tukey’s unequal N post hoc test for multiple comparisons. Categorical variables were compared using the Kruskal–Wallis test, followed by the post-hoc Dunn’s test for multiple comparisons. Correlations between selected variables and adiponectin, hs-CRP, and TNF-α levels were calculated using the Pearson correlation analysis for normally distributed variables or Spearman’s rank correlation for non-normally distributed variables. A simple logistic regression model was created to identify independent factors which increase the selection risk of MCI in the oldest group of patients with T2DM. Finally, the risk factors associated with MCI were identified using stepwise multivariable logistic regression (backward elimination with Wald criteria). Relevant odds ratios (OR) and 95% interval of confidence intervals (CI) were also computed. A *p*-value of less than 0.05 was considered statistically significant. The statistical analysis was performed with Statistica 13.1 (StatSoft, Krakow, Poland).

## 5. Conclusions

Our findings indicate that the participants with both MCI and depressive symptoms were characterized by the highest concentrations of hs-CRP and TNF-α and the lowest levels of adiponectin. Compared to controls without MCI or depression, these subjects tended to be older, less educated, female, and single with a longer duration of diabetes, higher number of co-morbidities, higher BMI and GDS-30 scores, higher HbA1c and triglyceride levels, and lower HDL cholesterol concentrations and MoCA scores. They were also more likely to be diagnosed with CVD, stroke/TIA, and hyperlipidemia, treated with insulin, and less likely to be receiving oral anti-diabetic drugs. Multivariate logistic regression indicated that factors associated with MCI in elderly diabetic patients were higher TNF-α levels, fewer years of formal education, increased number of co-morbidities, and the presence of CVD. Our findings also suggest that serum TNF-α can be a serological marker for MCI in elderly subjects with T2DM. However, further larger longitudinal studies are needed to better establish the role of adiponectin in regulating mood or cognition in elderly patients and its utility as a potential therapeutic target.

The elderly population is growing fast, and treatment requires a better understanding of individual needs and problems. In particular, the growth of dementia, cognitive impairment and depression at a very old age presents new challenges for the public health system. As such, there is a need for more strategies aimed at identifying and slowing the factors associated with disease progression to optimize complex health care while respecting the wishes and priorities of the patient.

## Figures and Tables

**Table 1:** Baseline clinical characteristics and metabolic parameters of elderly patients with T2DM.

	MCI n = 48	MCI with Depressive Symptoms n = 21	Depressive Symptoms without MCI n = 35	Controls n = 74
Age (years) *	84.4 ± 2.62 ^a^	87.6 ± 4.0 ^a,d,e^	83.5 ± 2.7 ^d^	84.0 ± 3.5 ^e^
Education-years *	9.68 ± 1.9 ^b,c^	9.28 ± 1.7 ^d,e^	12.1 ± 2.5 ^b,d^	12.1 ± 2.1 ^c,e^
Female sex (%) *	27 (56.3%) ^b^	17 (80.9%) ^e^	32 (91.4%) ^b,f^	27 (36.5%) ^e,f^
Partner status/living single (%) *	19 (39.5%) ^a^	18 (85.7%) ^a,e^	22 (62.8%)	25 (33.8%) ^e^
Current smokers (%) *	8 (16.7%) ^b^	11 (52.4%)	22 (62.8%) ^b,f^	18 (24.3%) ^f^
No physical activity (%) *	8 (16.7%) ^b^	11 (52.4%)	27 (77.1%) ^b,f^	24 (32.4%) ^f^
BMI (kg/m^2^) *	29.6 ± 3.37 ^a^	31.7 ± 3.4 ^a,e^	32.3 ± 3.7 ^f^	28.7 ± 3.1 ^e,f^
Time since diagnosis of diabetes (years) *	18.5 ± 6.3 ^c^	21.2 ± 6.54 ^e^	18.4 ± 7.7 ^f^	14.5 ± 4.4 ^c,e,f^
Glycemic control				
HbA1c (%) *	7.59 ± 0.6 ^b,c^	8.03 ± 0.7 ^d,e^	7.12 ± 0.6 ^b,d^	6.9 ± 0.5 ^c,e^
Lipids				
Total cholesterol (mmol/L)*	4.7 ± 1.0 ^b^	4.9 ± 0.96	5.58 ± 0.9 ^b,f^	4.47 ± 0.82 ^f^
Triglycerides (mmol/L) *	2.1 ± 0.5 ^b,c^	2.29 ± 0.5 ^d,e^	1.84 ± 0.3 ^b,d^	1.88 ± 0.34 ^c,e^
LDL (mmol/L) *	2.66 ± 0.64 ^b^	3.05 ± 0.85	3.38 ± 0.8 ^b,f^	2.61 ± 0.72 ^f^
HDL (mmol/L) *	1.08 ± 0.25 ^b,c^	1.00 ± 0.2 ^d,e^	1.24 ± 0.18 ^b,d^	1.21 ± 0.21 ^c,e^
Macrovascular complications Previous CVD *	26 (54.2%) ^c^	15 (71.4%) ^d,e^	8 (22.8%) ^d^	17 (22.9%) ^c,e^
Stroke/TIA *	1 (2.1%) ^a^	4 (19.1%) ^a,d,e^	2 (5.7%) ^d^	2 (2.7%) ^e^
Hyperlipidemia*	43 (89.6%) ^c^	21 (100%) ^e^	32 (91.4%) ^f^	50 (67.6%) ^c,e,f^
Retinopathy (%) *	33 (68.7%) ^b,c^	11 (52.4%)	11 (31.4%) ^b^	28 (37.8%) ^c^
Polyneuropathy (%) *	7 (14.6%)	7 (33.3%)	15 (42.8%) ^f^	6 (8.1%) ^f^
Nephropathy (%) *	28 (58.3%) ^c^	9 (42.8%)	10 (28.6%)	21 (28.4%) ^c^
Number of comorbidities *	6.31 ± 2.98 ^a,c^	9.19 ± 2.67 ^a,d,e^	5.14 ± 2.55 ^d,f^	3.05 ± 2.11 ^c,e,f^
Pharmacological therapy: Oral anti-diabetic drugs (%) *	47(97.2%) ^a,b^	6 (28.6%) ^a,e^	14 (40%) ^b,f^	71 (95.9%) ^e,f^
Insulin (%) *	16 (33.3%) ^a,b^	18 (85.7%) ^a,e^	26 (74.3%) ^b,f^	26 (35.1%) ^e,f^
Depressive symptoms (GDS score) *	3.39 ± 2.6 ^a,b^	15.8 ± 2.9 ^a,e^	15.6 ± 2.9 ^b,f^	2.89 ± 2.8 ^e,f^
MoCA score *	21.4 ± 1.45 ^b,c^	21.9 ± 1.74 ^d,e^	27.5 ± 1.63 ^b,d^	27.5 ± 1.17 ^c,e^

Abbreviations: T2DM—diabetes type 2, MCI—mild cognitive impairment, BMI—body mass index, HbA1c—glycosylated haemoglobin, LDL—low-density lipoprotein cholesterol, HDL—high-density lipoprotein cholesterol, CVD—cardiovascular disease, TIA—transient ischemic attack, GDS—Geriatric Depression Scale, and MoCA—Montreal Cognitive Assessment. Values are presented as mean ± standard deviations or percentages. * significant difference in ANOVA test (*p* < 0.05). ^a^ compare MCI group to MCI with depressive symptoms. ^b^ compare MCI group to depressive symptoms without MCI. ^c^ compare MCI group to controls. ^d^ compare MCI with depressive symptoms to depressive symptoms without MCI. ^e^ compare MCI with depressive symptoms to controls. ^f^ compare depressive symptoms without MCI to controls.

**Table 2:** Analysis of the correlation between selected quantitative variables (e.g., adiponectin, hs-CRP, and TNF-α level) in elderly patients with T2DM.

Parameter	Adiponectin r	*p*	hs-CRP r	*p*	TNF-α r	*p*
MCI (n = 48)						
HbA1c (%)	-	-	0.43	0.002	0.48	0.001
Adiponectin (µg/mL)					−0.36	0.01
hs-CRP (ng/mL)					0.42	0.003
TNF-α (pg/mL)	−0.36	0.01	0.42	0.003		
BMI (kg/m^2^)	−0.77	<0.001				
MoCA score			−0.57	<0.001	−0.58	<0.001
MCI with depressive symptoms (n = 21)						
HbA1c (%)	−0.77	<0.001	0.84	<0.001	0.59	0.004
Adiponectin (µg/mL)			−0.94	<0.001	-0.73	<0.001
hs-CRP (ng/mL)	−0.94	<0.001			0.69	0.001
TNF-α (pg/mL)	−0.73	<0.001	0.69	0.001		
Triglycerides (mmol/L)	−0.53	0.013	0.48	0.03	0.48	0.027
HDL (mmol/L)	0.46	0.03			−0.57	0.007
BMI (kg/m^2^)					0.77	<0.001
GDS-30 score	−0.77	<0.001	0.76	<0.001		
MoCA score	0.7	<0.001	−0.72	<0.001		
depressive symptoms without MCI (n = 35)						
HbA1c (%)					0.38	0.02
Adiponectin (µg/mL)			−0.42	0.014		
hs-CRP (ng/mL)	−0.42	0.014				
TNF-α (pg/mL)	−0.52	0.001	0.64	<0.001		
Total cholesterol (mmol/L)			0.34	0.04	0.59	<0.001
LDL (mmol/L)					0.35	0.03
BMI (kg/m^2^)	−0.85	<0.001			0.39	0.02
GDS-30 score	−0.52	0.001	0.6	<0.001	0.75	<0.001
MoCA score	0.49	0.002			−0.51	0.002
Controls n = 74						
Adiponectin (µg/mL)			−0.32	0.006	−0.33	0.003
hs-CRP (ng/mL)	−0.32	0.006			0.53	<0.001
TNF-α (pg/mL)	−0.33	0.003	0.53	<0.001		
Total cholesterol (mmol/L)			0.46	<0.001	0.38	0.001
LDL (mmol/L)			0.42	<0.001	0.31	0.007
BMI (kg/m^2^)	−0.7	<0.001	0.32	0.005	0.43	<0.001

r—correlation coefficient, *p*—significance. Abbreviations: T2DM—diabetes type 2, MCI—mild cognitive impairment, BMI—body mass index, HbA1c—glycosylated haemoglobin, LDL—low-density lipoprotein cholesterol HDL—high-density lipoprotein cholesterol, GDS—Geriatric Depression Scale, MoCA—Montreal Cognitive Assessment.

**Table 3:** Univariate and multivariate logistic regression analysis of risk factors for MCI in elderly patients with T2DM.

Parameter	ß	SE of ß	OR	95% CI	*p* Value
Univariate logistic regression analysis					
Age (years) *	0.14	0.04	1.15	1.04–1.25	0.004
Education-years *	−0.68	0.11	0.5	0.4–0.62	<0.001
Female sex	0.4	0.31	1.49	0.8–2.76	0.21
BMI (kg/m^2^)	0.05	0.04	1.03	0.95–1.12	0.44
Time since diagnosis of diabetes (years) *	0.09	0.02	1.1	1.04–1.16	0.001
HbA1c (%) *	1.65	0.28	5.23	3.01–9.09	<0.001
Total cholesterol (mmol/L)	0.02	0.004	1.0	0.99–1.01	0.63
Triglycerides (mmol/L) *	0.02	0.005	1.02	1.01–1.03	<0.001
LDL (mmol/L)	0.03	0.005	1.0	0.98–1.02	0.5
HDL (mmol/L) *	−0.08	0.02	0.92	0.88–0.96	<0.001
Adiponectin (µg/mL) *	−0.29	0.04	0.75	0.68–0.82	<0.001
hs-CRP (ng/mL) *	0.81	0.13	2.24	1.72–2.91	<0.001
TNF-α (pg/mL) *	0.82	0.12	2.26	1.79–2.86	<0.001
Previous CVD *	1.59	0.34	4.92	2.55–9.48	<0.001
Stroke/TIA	0.71	0.68	2.05	0.53–7.92	0.29
Hypertension	0.54	0.38	1.72	0.81–3.65	0.15
Hyperlipidemia *	1.44	0.52	4.22	1.54–11.5	0.005
Retinopathy *	1.15	0.32	3.16	1.68–5.91	<0.001
Polyneuropathy	0.06	0.38	1.06	0.5–2.27	0.86
Nephropathy *	1.07	0.32	2.9	1.54–5.46	<0.001
Depressive symptoms (GDS score)	0.05	0.02	1.01	0.96–1.05	0.82
Number of comorbidities *	0.4	0.06	1.49	1.31–1.7	<0.001
Multivariate logistic regression analysis					
TNF-α (pg/mL) *	0.72	0.14	2.05	1.55–2.69	<0.001
Education-years *	−0.46	0.13	0.63	0.48–0.82	0.001
Number of comorbidities *	0.18	0.08	1.21	1.03–1.42	0.022
Previous CVD *	1.42	0.53	4.15	1.46–11.81	0.008

* statistically significant difference, *p* < 0.05; ß: regression coefficient; CI: confidence interval for odds ratio; OR: odds ratio; SE: standard error. Abbreviations: T2DM—diabetes type 2, MCI—mild cognitive impairment, BMI—body mass index, HbA1c—glycosylated haemoglobin, LDL—low-density lipoprotein cholesterol HDL—high-density lipoprotein cholesterol, CVD—cardiovascular disease, TIA—transient ischemic attack, GDS—Geriatric Depression Scale, MoCA—Montreal Cognitive Assessment.

## Data Availability

Dataset available on request from the authors.

## References

[1] International Diabetes Federation (2021). IDF Diabetes Atlas.

[2] Centers for Disease Control and Prevention National Diabetes Statistics Report Website. https://www.cdc.gov/diabetes/php/data-research/index.html#cdc_report_pub_study_section_2-prevalence-of-both-diagnosed-and-undiagnosed-diabetes.

[3] Xue M., Xu W., Ou Y.N., Cao X.P., Tan M.S., Tan L., Yu J.T. (2019). Diabetes mellitus and risks of cognitive impairment and dementia: A systematic review and meta-analysis of 144 prospective studies. Ageing Res. Rev..

[4] Petersen R.C., Roberts R.O., Knopman D.S., Boeve B.F., Geda Y.E., Ivnik R.J., Smith G.E., Jack C.R. (2009). Mild cognitive impairment: Ten years later. Arch. Neurol..

[5] Farias S.T., Mungas D., Reed B.R., Harvey D., DeCarli C. (2009). Progression of mild cognitive impairment to dementia in clinic- vs community-based cohorts. Arch. Neurol..

[6] Nanayakkara N., Pease A., Ranasinha S., Wischer N., Andrikopoulos S., Speight J., de Courten B., Zoungas S. (2018). Depression and diabetes distress in adults with type 2 diabetes: Results from the Australian National Diabetes Audit (ANDA) 2016. Sci. Rep..

[7] Abdoli N., Salari N., Darvishi N., Jafarpour S., Solaymani M., Mohammadi M., Shohaimi S. (2022). The global prevalence of major depressive disorder (MDD) among the elderly: A systematic review and meta-analysis. Neurosci. Biobehav. Rev..

[8] Zhang H., Xing Y., Zhang Y., Sheng S., Zhang L., Dong Z., Gao Q., Cai W., Mou Z., Jing Q. (2023). Association between depression and quality of life in older adults with type 2 diabetes: A moderated mediation of cognitive impairment and sleep quality. J. Affect. Disord..

[9] van Sloten T.T., Sedaghat S., Carnethon M.R., Launer L.J., Stehouwer C.D.A. (2020). Cerebral microvascular complications of type 2 diabetes: Stroke, cognitive dysfunction, and depression. Lancet Diabetes Endocrinol..

[10] Roberts R.O., Geda Y.E., Knopman D.S., Cha R.H., Boeve B.F., Ivnik R.J., Pankratz V.S., Tangalos E.G., Petersen R.C. (2010). Metabolic syndrome, inflammation, and nonamnestic mild cognitive impairment in older persons: A population-based study. Alzheimer Dis. Assoc. Disord..

[11] Maydych V. (2019). The Interplay Between Stress, Inflammation, and Emotional Attention: Relevance for Depression. Front. Neurosci..

[12] Gorska-Ciebiada M., Saryusz-Wolska M., Borkowska A., Ciebiada M., Loba J. (2015). Serum levels of inflammatory markers in depressed elderly patients with diabetes and mild cognitive impairment. PLoS ONE.

[13] Zambon A., Pauletto P., Crepaldi G. (2005). Review article: The metabolic syndrome--a chronic cardiovascular inflammatory condition. Aliment. Pharmacol. Ther..

[14] Takahashi M., Oda Y., Sato K., Shirayama Y. (2018). Vascular risk factors and the relationships between cognitive impairment and hypoperfusion in late-onset Alzheimer’s disease. Acta Neuropsychiatr..

[15] Kadowaki T., Yamauchi T., Kubota N., Hara K., Ueki K., Tobe K. (2006). Adiponectin and adiponectin receptors in insulin resistance, diabetes, and the metabolic syndrome. J. Clin. Investig..

[16] Letra L., Santana I., Seica R. (2014). Obesity as a risk factor for Alzheimer’s disease: The role of adipocytokines. Metab. Brain Dis..

[17] Thundyil J., Pavlovski D., Sobey C.G., Arumugam T.V. (2012). Adiponectin receptor signalling in the brain. Br. J. Pharmacol..

[18] Rizzo M.R., Fasano R., Paolisso G. (2020). Adiponectin and Cognitive Decline. Int. J. Mol. Sci..

[19] Bloemer J., Pinky P.D., Govindarajulu M., Hong H., Judd R., Amin R.H., Moore T., Dhanasekaran M., Reed M.N., Suppiramaniam V. (2018). Role of Adiponectin in Central Nervous System Disorders. Neural. Plast..

[20] Kim K.Y., Ha J., Kim M., Cho S.Y., Kim H., Kim E., Alzheimer’s Disease Neuroimaging Initiative (2022). Plasma adiponectin levels predict cognitive decline and cortical thinning in mild cognitive impairment with beta-amyloid pathology. Alzheimer’s Res. Ther..

[21] Hall A., Pekkala T., Polvikoski T., van Gils M., Kivipelto M., Lötjönen J., Mattila J., Kero M., Myllykangas L., Mäkelä M. (2019). Prediction models for dementia and neuropathology in the oldest old: The Vantaa 85+ cohort study. Alzheimer’s Res. Ther..

[22] Chen J.F., Zhang Y.P., Han J.X., Wang Y.D., Fu G.F. (2023). Systematic evaluation of the prevalence of cognitive impairment in elderly patients with diabetes in China. Clin. Neurol. Neurosurg..

[23] Brzezińska A., Bourke J., Rivera-Hernández R., Tsolaki M., Woźniak J., Kaźmierski J. (2020). Depression in Dementia or Dementia in Depression? Systematic Review of Studies and Hypotheses. Curr. Alzheimer Res..

[24] Steffens D.C., McQuoid D.R., Payne M.E., Potter G.G. (2011). Change in hippocampal volume on magnetic resonance imaging and cognitive decline among older depressed and nondepressed subjects in the neurocognitive outcomes of depression in the elderly study. Am. J. Geriatr. Psychiatry.

[25] Jamieson A., Goodwill A.M., Termine M., Campbell S., Szoeke C. (2019). Depression related cerebral pathology and its relationship with cognitive functioning: A systematic review. J. Affect. Disord..

[26] Du Y., Zhang Q., Zhang X., Song Y., Zheng J., An Y., Lu Y. (2023). Correlation between inflammatory biomarkers, cognitive function and glycemic and lipid profiles in patients with type 2 diabetes mellitus: A systematic review and meta-analysis. Clin. Biochem..

[27] Nguyen M.M., Perlman G., Kim N., Wu C.Y., Daher V., Zhou A., Mathers E.H., Anita N.Z., Lanctôt K.L., Herrmann N. (2021). Depression in type 2 diabetes: A systematic review and meta-analysis of blood inflammatory markers. Psychoneuroendocrinology.

[28] Mehdi S., Wani S.U.D., Krishna K.L., Kinattingal N., Roohi T.F. (2023). A review on linking stress, depression, and insulin resistance via low-grade chronic inflammation. Biochem. Biophys. Rep..

[29] Assar M.E., Angulo J., Rodríguez-Mañas L. (2016). Diabetes and ageing-induced vascular inflammation. J. Physiol..

[30] Espeland M.A., Erickson K., Neiberg R.H., Jakicic J.M., Wadden T.A., Wing R.R., Desiderio L., Erus G., Hsieh M.K., Davatzikos C. (2016). Brain and White Matter Hyperintensity Volumes after 10 Years of Random Assignment to Lifestyle Intervention. Diabetes Care.

[31] Triviño-Paredes J., Patten A.R., Gil-Mohapel J., Christie B.R. (2016). The effects of hormones and physical exercise on hippocampal structural plasticity. Front. Neuroendocrinol..

[32] Hu Y., Dong X., Chen J. (2015). Adiponectin and depression: A meta-analysis. Biomed. Rep..

[33] Platzer M., Fellendorf F.T., Bengesser S.A., Birner A., Dalkner N., Hamm C., Hartleb R., Queissner R., Pilz R., Rieger A. (2019). Adiponectin is decreased in bipolar depression. World J. Biol. Psychiatry.

[34] Cao B., Chen Y., Brietzke E., Cha D., Shaukat A., Pan Z., Park C., Subramaniapillai M., Zuckerman H., Grant K. (2018). Leptin and adiponectin levels in major depressive disorder: A systematic review and meta-analysis. J. Affect. Disord..

[35] Huang C., Kogure M., Tomata Y., Sugawara Y., Hozawa A., Momma H., Tsuji I., Nagatomi R. (2020). Association of serum adiponectin levels and body mass index with worsening depressive symptoms in elderly individuals: A 10-year longitudinal study. Aging Ment. Health..

[36] Herder C., Schmitt A., Budden F., Reimer A., Kulzer B., Roden M., Haak T., Hermanns N. (2018). Association between pro- and anti-inflammatory cytokines and depressive symptoms in patients with diabetes-potential differences by diabetes type and depression scores. Transl. Psychiatry.

[37] Liu Z.Q., Zhang M.X., Wang J., Ding N. (2017). Analysis of correlation between the mild cognitive impairment (MCI) and level of adiponectin in elderly patients with type 2 diabetes mellitus (T2DM). Eur. Rev. Med. Pharmacol. Sci..

[38] Liu H., Ma J., Sun L., Zhang Q., Fan J. (2021). Relationship between cognitive impairment and serum amyloid β-protein, adiponectin, and C-reactive protein levels in type II diabetes patients. Ann. Palliat. Med..

[39] Teixeira A.L., Diniz B.S., Campos A.C., Miranda A.S., Rocha N.P., Talib L.L., Gattaz W.F., Forlenza O.V. (2013). Decreased levels of circulating adiponectin in mild cognitive impairment and Alzheimer’s disease. Neuromolecular. Med..

[40] Une K., Takei Y.A., Tomita N., Asamura T., Ohrui T., Furukawa K., Arai H. (2011). Adiponectin in plasma and cerebrospinal fluid in MCI and Alzheimer’s disease. Eur. J. Neurol..

[41] Nasreddine Z.S., Phillips N.A., Bédirian V., Charbonneau S., Whitehead V., Collin I., Cummings J.L., Chertkow H. (2005). The Montreal Cognitive Assessment, MoCA: A brief screening tool for mild cognitive impairment. J. Am. Geriatr. Soc..

[42] Alagiakrishnan K., Zhao N., Mereu L., Senior P., Senthilselvan A. (2013). Montreal Cognitive Assessment is superior to Standardized Mini-Mental Status Exam in detecting mild cognitive impairment in the middle-aged and elderly patients with type 2 diabetes mellitus. BioMed Res. Int..

[43] Ciesielska N., Sokołowski R., Mazur E., Podhorecka M., Polak-Szabela A., Kędziora-Kornatowska K. (2016). Is the Montreal Cognitive Assessment (MoCA) test better suited than the Mini-Mental State Examination (MMSE) in mild cognitive impairment (MCI) detection among people aged over 60? Meta-analysis. Psychiatr. Pol..

[44] Portet F., Ousset P.J., Visser P.J., Frisoni G.B., Nobili F., Scheltens P., Vellas B., Touchon J., MCI Working Group of the European Consortium on Alzheimer’s Disease (EADC) (2006). Mild cognitive impairment (MCI) in medical practice: A critical review of the concept and new diagnostic procedure. Report of the MCI Working Group of the European Consortium on Alzheimer’s Disease. J. Neurol. Neurosurg. Psychiatry.

[45] Yesavage J.A., Brink T.L., Rose T.L., Lum O., Huang V., Adey M., Leirer V.O. (1982). Development and validation of a geriatric depression screening scale: A preliminary report. J. Psychiatr. Res..

